# Development and comparison of a minimally-invasive model of autologous clot pulmonary embolism in Sprague-Dawley and Copenhagen rats

**DOI:** 10.1186/1477-9560-8-3

**Published:** 2010-02-11

**Authors:** Michael S Runyon, Michael A Gellar, Nina Sanapareddy, Jeffrey A Kline, John A Watts

**Affiliations:** 1 Emergency Medicine Research, Carolinas Medical Center, Charlotte, NC, USA; 2 Department of Bioinformatics, University of North Carolina Charlotte, Charlotte, NC, USA

## Abstract

**Background:**

Experimental models of pulmonary embolism (PE) that produce pulmonary hypertension (PH) employ many different methods of inducing acute pulmonary occlusion. Many of these models induce PE with intravenous injection of exogenous impervious objects that may not completely reproduce the physiological properties of autologous thromboembolism. Current literature lacks a simple, well-described rat model of autlogous PE. Objective: Test if moderate-severity autologous PE in Sprague-Dawley (SD) and Copenhagen (Cop) rats can produce persistent PH.

**Methods:**

blood was withdrawn from the jugular vein, treated with thrombin-Ca^++ ^and re-injected following pretreatment with tranexamic acid. Hemodynamic values, clot weights and biochemical measurements were performed at 1 and 5 days.

**Results:**

Infusion of clot significantly increased the right ventricular peak systolic pressure to 45-55 mm Hg, followed by normalization within 24 hours in SD rats, and within 5 days in COP rats. Clot lysis was 95% (24 hours) and 97% (5 days) in SD rats and was significantly lower in COP rats (70%, 24 hours; 87% 5 days). Plasma D-dimer was elevated in surgical sham animals and was further increased 8 hours after pulmonary embolism. Neither strain showed a significant increase in bronchoalveolar chemotactic activity, myeloperoxidase activity, leukocyte infiltration, or chemokine accumulation, indicating that there was no significant pulmonary inflammation.

**Conclusions:**

Both SD and COP rats exhibited near complete fibrinolysis of autologous clot PE within 5 days. Neither strain developed persistent PH. Experimental models of PE designed to induce sustained PH and a robust inflammatory response appear to require significant, persistent pulmonary vascular occlusion.

## Background

Several laboratory models have been developed and implemented to study experimental pulmonary embolism (PE) in the rat. In the broadest terms, these models can be categorized into two groups: injected thrombus versus foreign body. The thrombus models utilize a blood sample obtained from the same (autologous) or a donor (heterologous) animal that is coagulated ex-vivo to produce an organized thrombus which is then (re)injected into the experimental animals [1]. The foreign body models infuse inert objects, such as polystyrene, sephadex or glass microspheres, into the venous circulation to create a fixed pulmonary vascular obstruction [2-4]. Each method offers advantages and limitations. The injected clot model provides a closer representation of acute pulmonary thromboembolism, but the percentage and persistence of the resultant pulmonary vascular occlusion can be difficult to control because of the irregular size and volume of ex-vivo clots, and the effect of the rat's high rate of fibrinolysis. The physiologic effects of hemolysis associated with clot formation and injection represent another potential confounding variable in such models. Additionally, although blood for thrombus can be donated from a highly genetically similar littermate, this does not exclude the possibility of a mild immunological reaction. Existing autologous models of PE require a two-stage laparatomy[5], or produce profound circulatory shock in rats[1], rabbits[6] or pigs[7] On the other hand, foreign body models can be more carefully titrated to control the severity of PE, but it is self-evident that plastic or glass beads incompletely reproduce the pathophysiologic manifestations of acute pulmonary thromboembolism.

We sought to develop a less invasive rat model of moderately severe autologous-clot PE. Many of the previously cited models utilize Sprague-Dawley rats, a strain shown to demonstrate high rates of endogenous fibrinolytic activity [8]. However, work by Cooley, et al, has revealed a potentially profound prothrombotic tendency in Copenhagen rats [9]. Therefore, we performed our experiments in both Sprague-Dawley and Copenhagen rats and compared the effects of our model among the two strains.

## Materials and methods

All experiments were conducted in accordance with the National Institutes of Health guidelines on the use of experimental animals. The study protocol was approved by the Carolinas Medical Center Institutional Animal Care and Use Committee and all experiments were conducted in accordance with the Guide for the Care and Use of Laboratory Animals. Animals were housed in an Association for Assessment and Accreditation of Laboratory Animal Care-approved level III vivarium and allowed ad libitum access to food and water prior to all procedures and after recovery from anesthesia. Studies were conducted in male Sprague-Dawley and Copenhagen rats weighing 366-429 grams and 220-266 grams, respectively.

Following anesthesia with an intraperitoneal injection of a mixture of xylazine (3 mg/kg) and ketamine (70 mg/kg), the animals were weighed, a baseline temperature measurement taken, and they were placed on a warming pad filled with re-circulating water warmed to 105°F (Gaymar solid-state T-pump; Orchard Park, NY). The neck was shaved and the skin prepped aseptically. The right jugular vein was dissected and cannulated with a 2-Fr Millar Mikro-Tip catheter transducer (Millar Instruments, Houston, TX). The Millar transducers were connected to a Transducer Balance Box, a UM 100A coupling box and an MP100 data acquisition unit, which was connected to a personal computer. Pressures were recorded using Acknowledge software (BIOPAC Systems, Goleta, CA). The transducer was advanced into the right ventricle for baseline pressure measurement. The transducer was removed and the vein cannulated with PE-50 tubing and 0.5 mL blood withdrawn into a heparinized syringe for D-dimer measurement (initial D-dimer, -24 hr). PE-200 (Sprague-Dawley) or PE-160 (Copenhagen) tubing was then inserted into the right external jugular vein and blood withdrawn to fill the length of the tubing and just into the barrel of a 12 mL syringe that was been pre-filled with 100 μL calcium chloride (100 mM) and 100 μL thrombin (10 units/mL) to enhance clot formation. The length of the tubing, and the resultant volume of clot, were weight-based and took into account the relative smaller size of the Copenhagen animals. Pilot data determined the optimal total clot dose to reliably result in acute pulmonary hypertension, defined as a right ventricular pressure of greater than 40 mmHg, to be 22 mg per 100 grams body weight for the Sprague-Dawley animals and significantly less, 2.8 mg per 100 grams body weight, for the Copenhagen animals. The catheter was removed from the animal and the blood drawn completely into the syringe and agitated to facilitate mixing with the calcium and thrombin before being injected back into the tubing with care taken to avoid air bubbles. The clot was incubated in the tubing at 37°C for 3 hours and then placed at 4°C for 21 hours. Blood loss was replaced with a volume of 0.9% sodium chloride equal to two and one half times that of the blood removed. The skin incision was closed with a surgical clip and the animal was allowed to recover from anesthesia.

**Figure 1:**
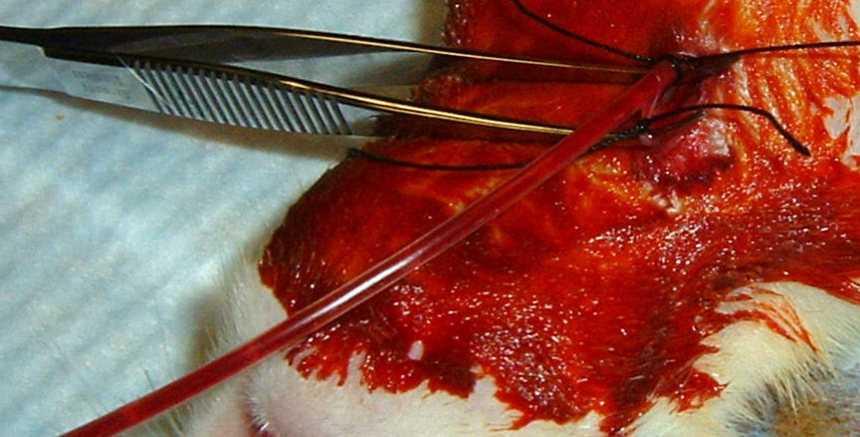
**Tubing with segmented, "hardened" clot material just before injection into the jugular vein of a rat**.

**Figure 2:**
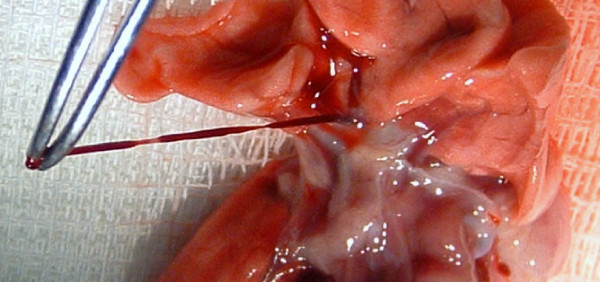
**Segmented "hardened" clot material retrieved from the pulmonary artery immediately after injection into the jugular vein of a rat, illustrating the embolization of injected clot into the lung vasculature**.

At 24 hours, the clot-containing tubing was retrieved and the distal end disinfected with 2% chlorhexidine diacetate and rinsed with sterile water. The animals were anesthetized and prepped as above, the wound clip removed, and the incision opened. The right jugular vein was re-cannulated, the right ventricular pressure measured, and 0.5 mL of blood was drawn into a heparinized syringe for D-dimer measurement (0 hr) (Asserachrom D-Dimer kit, Diagnostica, Stago, Parsippany, NJ). To attenuate the animal's endogenous fibrinolytic activity, tranexamic acid was injected (60 mg/100 gm) intravenously prior to clot injection. Tranexamic acid has been shown to increase the persistence of embolic clot material in rats [10]. The distal one cm of the clot-containing tubing was amputated and discarded, the newly cut end of the tubing was inserted into the right jugular vein, and the clot was segmented by crimping the tubing with hemostats at 1 cm intervals (Figures 1 &2). To minimize mortality during clot injection, the initial clot bolus (18 mg/100 grams body weight for Sprague-Dawley and 5 mg for Copenhagen animals) was infused slowly over five minutes. The tubing was then removed and the right ventricular pressure measured. If the right ventricular pressure was less than 40 mmHg, a supplemental clot dose (4 mg/100 grams body weight for Sprague-Dawley and up to 5 mg for Copenhagen animals) was infused over five minutes. If the right ventricular pressure remained less than 40 mmHg after the supplemental clot injection, the animal was removed from the study and euthanized with an intraperitoneal injection of 260 mg/kg of 26% sodium pentobarbital. Animals meeting the targeted right ventricular pressure had an indwelling tunneled catheter placed in the jugular vein to facilitate interval blood draws for D-dimer measurement at 8 hours and 24 hours after clot injection. The animals were then returned to their cages and allowed to recover. The sham procedure was the same as above, except that the clotted blood was discarded and 0.9% NaCl was infused into the animal instead of the clot.

At the end of the experiment (24 hours or 5 days), the animals were anesthetized and prepped as above. The carotid artery was dissected and a 2-Fr Millar Mikro-Tip catheter transducer inserted to measure arterial pressure. The right ventricular pressure was measured as previously described.

Pleural effusions were collected, if present, and pleural wash and bronchoalveolar lavage (BAL) performed as previously described [11]. The recovered fluid was centrifuged and the supernatant was stored at -70°F. The cell pellets were subjected to hypotonic cell lysis to eliminate erythrocytes and leukocyte infiltration quantified on a hemocytometer. After centrifugation, the cell pellets were stored at -70°F until needed for additional measurements. The heart and lungs were removed and the pulmonary arteries dissected to retrieve any clot. Recovered clot was weighed (wet weight) and then dried for 24 hours at 37°C and re-weighed (dry weight). Clot lysis was estimated by subtracting the dry weight of the recovered clot from the estimated dry weight of the total injected clot dose.

### Chemotactic activity

Neutrophils were purified using Polymorphprep™ cell separation medium (Accurate Chemical and Scientific, Westbury, NY) and chemotaxis assays performed using 24-well Corning-Costar Transwell^® ^migration chambers as previously described [11]. Formaldehyde/EDTA-fixed cells were collected by centrifugation (5 min at 450 × *g*) and counted with a hemocytometer.

### Myeloperoxidase activity

Myeloperoxidase (MPO) activity was measured in BAL cell pellets that were suspended in lysis buffer (100 mM potassium phosphate [pH 6.0], 5 mM EDTA, and 0.5% hexadecyltrimethylammonium bromide). The assay was performed in 96-well microtiter plates by adding substrate buffer containing 0.01% *o*-dianisidine-HCl and 0.01% hydrogen peroxide. Plates were incubated at room temperature for one hour and read at 450 nm in a UV Max microplate reader (Molecular Devices, Sunnyvale, CA). Protein was quantified in each MPO extract using the biconchoninic acid (BCA) method and MPO activity was normalized to protein concentration. MPO standard curves were constructed using purified human myeloperoxidase (Sigma-Aldrich catalog no. M6908).

### Chemokine measurements

Chemokine protein content was determined in the BAL supernatant utilizing the following assays: Monocyte chemoattractant protein (MCP-1) (OptEIA set #555130 and reagent A set #55053, Pharmingen, San Diego, CA); Cytokine-induced neutrophil chemoattractant, CINC-1 and CINC-2 (DuoSet kits #DY515 and # DY516, R&D Systems, Minneapolis, MN); CINC-3 (Macrophage-inflammatory protein-2, created using mouse monoclonal anti-CINC3 primary mAb #MAB525, biotinylated goat anti-CINC3 secondary IgG R&D #BAF525 and recombinant rat CINC3 standard #525-C3, R&D Systems, Minneapolis, MN).

### Statistics

Data are shown as means ± standard error of the mean. Continuous data were tested for normality using the Wilk-Shapiro statistic (P > 0.1). Hemodynamic variables that were measured more than once in the same animals were compared using repeat measures ANOVA (RM ANOVA) followed by t-tests. Clot content, measured at the time of dosing and at the end of the experiment was tested by paired t-test and independent continuous variables were compared between groups with an unpaired t-test with P < 0.05 considered significant. We used a Bonferroni-adjusted P value to correct for multiple measurements.

## Results

The experimental animals consisted of 58 total animals, 40 Sprague-Dawley and 18 Copenhagen rats. We allocated 10 Sprague-Dawley animals to each of 4 groups: 24-hour PE, 24-hour sham, 5-day PE, and 5-day sham. We planned for 5 Copenhagen animals each in the same 4 groups, but there was one death in each of the PE groups, leaving 4 animals each of the 2 PE groups and 5 in each of the sham groups. Among each strain, the animals were similar weights, but between strains, the Copenhagen animals were significantly smaller (Table 1). This difference was expected given their respective normal growth curves.

**Table 1:** Lung inflammation measurements

Parameter	Time	Sprague-Dawley			Copenhagen		
		PE	Sham	**Sig**.	PE	Sham	Sig
Weight (g)	24 hr	399 ± 5	392 ± 4	n.s.	240 ± 8	254 ± 2	n.s.
	5 day	402 ± 5	393 ± 5	n.s.	244 ± 8	245 ± 4	n.s.
Pleural WBC (×10^5^)	24 hr	108 ± 19	109 ± 32	n.s.	27 ± 4	32 ± 4	n.s.
	5 day	107 ± 16	109 ± 4	n.s.	39 ± 8	31 ± 3	n.s.
BAL WBC (×10^3^)	24 hr	543 ± 45	337 ± 35	*	491 ± 26	415 ± 51	n.s.
	5 day	587 ± 82	514 ± 40	n.s.	449 ± 65	435 ± 44	n.s.
BAL Chemotaxis (×10^3^)	24 hr	52 ± 9	33 ± 3	n.s.	16 ± 1	17 ± 2	n.s.
	5 day	41 ± 4	37 ± 4	n.s.	18 ± 2	13 ± 5	n.s.
BAL MPO (U/mL)	24 hr	229 ± 79	104 ± 23	n.s.	128 ± 11	152 ± 57	n.s.
	5 day	256 ± 133	301 ± 65	n.s.	142 ± 87	102 ± 19	n.s.
BAL MCP-1 (pg/mL)	24 hr	137 ± 90	42 ± 14	n.s.	188 ± 112	134 ± 48	n.s.
	5 day	24 ± 13	101 ± 80	n.s.	187 ± 105	228 ± 59	n.s.
BAL CINC-1 (pg/mL)	24 hr	62 ± 32	32 ± 11	n.s.	66 ± 14	38 ± 12	n.s.
	5 day	21 ± 6	30 ± 12	n.s.	36 ± 8	41 ± 7	n.s.
BAL CINC-2 (pg/mL)	24 hr	638 ± 51	554 ± 32	n.s.	267 ± 23	220 ± 28	n.s.
	5 day	610 ± 45	552 ± 40	n.s.	266 ± 42	280 ± 18	n.s.
BAL CINC-3 (pg/mL)	24 hr	21 ± 1	21 ± 0.4	n.s.	19 ± 4	19 ± 8	n.s.
	5 day	20 ± 0.3	20 ± 0.1	n.s.	18 ± 6	15 ± 4	n.s.

Values are mean ± standard error, where n = 10 Sprague-Dawley, n = 4-5 Copenhagen. *Indicates a value significantly different from vehicle-treated Sham animals (Bonferroni-adjusted p < 0.05, t-Test). Abbreviations: not significant (n.s.); pleural white blood cell count (WBC); bronchoalveolar lavage fluid (BAL); white blood cell count (WBC); in vitro neutrophil chemotaxis in response to BAL fluid (Chemotaxis) myeloperoxidase activity (MPO); monocyte chemotactic factor (MCP); cytokine-induced neutrophil chemotactic factor (CINC).

### Sprague-Dawley Results

**Figure 3:**
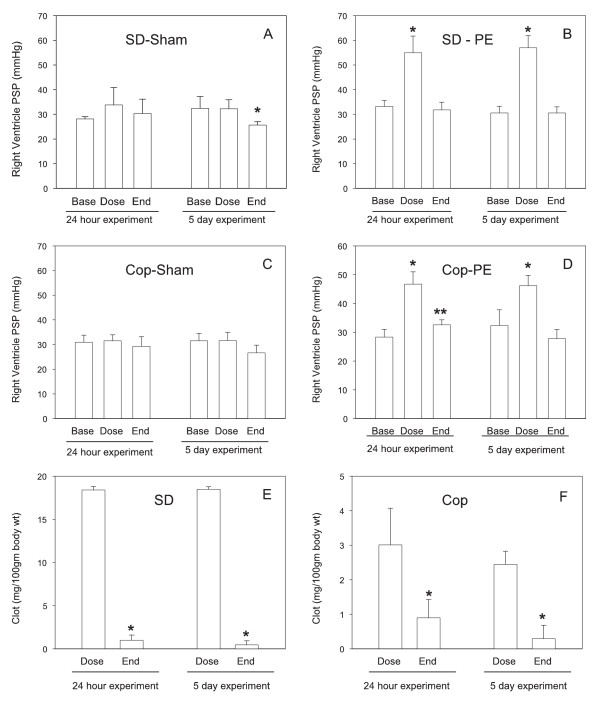
**Hemodynamic and clot content measured at the baseline (base), immediately after dosing with clot (Dose) and at the end of the 24 hour or 5 day experiment in Sprague-Dawley (SD) or Copenhagen (Cop) strains of rats**. Animals were treated with vehicle (Sham) or with pulmonary embolism (PE) injections. Values are mean ± standard error, where n = 10 in the SD and 4-5 in Cop experiments. *Significantly different from Base (RM ANOVA, t-Test). **Significantly different from Base and from Dose (RM ANOVA, t-Test).

**Figure 4:**
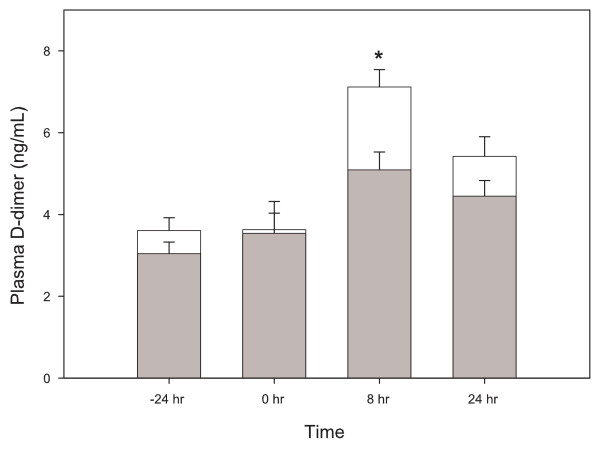
**Plasma D-dimer concentration measured before (-24 hr), and 8 hr and 24 hr after clot injection in Sprague-Dawley rats**. Grey bars are the sham animals and clear bars are the PE animals. Values were pooled from the 24 hour and 5 day experiment. Values are mean ± standard error, n = 20. Star indicates a value significantly higher than the sham value at the same time point.

The total clot dose for the Sprague-Dawley animals was 18.4 ± 0.1 mg/100 grams and 18.5 ± 0.1 mg/100 grams for the 24-hour and 5-day groups respectively (Figure 3). At the end of the experiments, a measurable amount of residual clot was retrieved from the pulmonary arteries of all the PE animals, save one in the 5-day group. Overall clot lysis was 95 ± 1.0% at 24 hours and 97 ± 0.8% at 5 days. There was no clot recovered from the pulmonary arteries of any of the sham animals. Plasma D-dimer values were increased significantly more 8 hours after injection of clot material compared with sham surgery and returned to sham levels by 24 hours after injection of clot material in the Sprague-Dawley PE group (Figure 4).

Baseline right ventricular pressures, measured before treatment, were not significantly different (p > 0.05 all comparisons) in any of the experimental groups (Figure 3). Peak right ventricular pressure increased significantly following clot injection (55.0 ± 2.1 mmHg clot versus 33.2 ± 0.8 baseline, 24 hr experiment; 57.0 ± 1.6 clot versus 30.6 ± 0.9 baseline, 5 day experiment), while animals receiving sham injection showed no significant change in peak right ventricular pressure in either experiment. Right ventricular pressures returned to values not significantly different from baseline 24 hours or 5 days after induction of PE (Figure 3). Due to technical difficulty with inserting the pressure transducing catheter, right ventricular pressure measurements were unobtainable in one animal in the 24-hour PE group and in 3 each in the 5-day PE and 5-day sham groups.

Several indices of inflammation were examined in the Sprague-Dawley rat lungs (Table 1). Chemotactic activity of the BAL supernatant was not different between sham and PE animals in either the 24 hour or 5 day experiment. Examination of the BAL cell pellet demonstrated no significant difference in myeloperoxidase activity among the 4 groups. BAL pellet cell counting revealed 543 ± 45.1 × 10^3 ^and 337 ± 35.4 × 10^3 ^leukocytes in the 24-hour PE and sham (p = 0.002), and 587 ± 82.4 × 10^3 ^and 514 ± 39.5 × 10^3 ^leukocytes in the 5-day PE and sham animals. Measured levels of the chemokines MCP-1, CINC-1, CINC-2, and CINC-3 are shown in Table 1 and were not significantly different in the PE versus sham animals at either time point.

### Copenhagen Results

The total clot dose for the Copenhagen animals was 3.0 ± 0.5 mg/100 grams and 2.4 ± 0.2 mg/100 grams for the 24-hour and 5-day groups respectively (Figure 3). At the end of the experiments, residual clot was retrieved from the pulmonary arteries of all the experimental animals, except for two in the 5-day group. Overall clot lysis was 69.8 ± 7.4% at 24 hours (p < 0.001 compared to the 24-hour Sprague-Dawley PE animals) and 87.3 ± 7.6% at 5 days (p < 0.052 compared to the 5-day Sprague-Dawley PE animals). There was no clot recovered from the pulmonary arteries of any of the sham animals.

Baseline right ventricular pressure, measured before treatment, were not significantly different (p > 0.05 all comparisons) in any of the experimental groups (Figure 3). Peak right ventricular pressure increased significantly following clot injection (44.9 ± 1.5 mmHg clot versus 27.0 ± 0.9 baseline in the 24-hour experiment, and 44.7 ± 1.5 clot versus 31.8 ± 1.8 baseline in the 5-day experiment, while animals receiving sham injection showed no significant change in right ventricular pressure in either experiment. The right ventricular pressure partially returned to baseline (32.0 ± 1.0 mmHg PE 24 hr versus 27.0 ± 0.9 mmHg baseline, p < 0.007) at the end of the 24-hour experiment and was not significantly different from baseline at 5 days. Due to technical difficulty with inserting the pressure transducer, right ventricular pressure measurements were unobtainable in one animal in the 24-hour PE group and in 2 in the 5-day sham group.

Inflammatory indices measured in the Copenhagen animals are shown in Table 1. Chemotactic activity of the BAL supernatant was not different between sham and PE animals in either the 24 hour or 5 day experiment. Examination of the BAL cell pellet demonstrated no difference in myeloperoxidase activity among the 4 groups. BAL pellet cell counting revealed 491 ± 25.6 × 10^3 ^and 415 ± 51.5 × 10^3 ^leukocytes in the 24-hour PE and sham, and 449 ± 65.1 × 10^3 ^and 435 ± 43.6 × 10^3 ^leukocytes in the 5-day PE and sham animals. Measured levels of the chemokines MCP-1, CINC-1, CINC-2, and CINC-3 were not significantly different in the PE versus sham animals at either time point.

## Discussion

The main findings of this work was that neither Sprague Dawley nor Copenhagen rat strains demonstrated sustained elevations in right ventricular pressures or a robust lung inflammatory response after PE induced by autologous clot in the presence of tranexamic acid. These findings contrast to the pronounced increases in right ventricular pressure and lung inflammatory responses observed after hilar clamp-release models [12], bead-induced fixed lung ischemia [11], and chemical-induced pulmonary hypertension [13]. These observations suggest that persistent vascular obstruction sufficient to result in sustained pulmonary hypertension appears necessary to induce the inflammatory response.

Multiple indices of lung inflammation were examined in the present studies, including BAL leukocyte content, chemotactic activity, myeloperoxidase activity and chemokine content. There was very little indication of stimulation of lung inflammatory processes in any of these measurements following the infusion of clot material in either strain of rat. These indications were limited to a significant increase in BAL WBC count and non significant elevation in values for BAL chemotaxis, MPO and MCP-1 in Sprague-Dawley rats (Table 1). Persistent obstruction and lung inflammation were not obtained using single administration of autologous clot material in either the Sprague-Dawley or Copenhagen rats. A potential limitation of the present studies is that recurrent delivery of clot material was not studied. In contrast, our previous studies with fixed pulmonary vascular obstruction using polystyrene microspheres in Sprague-Dawley rats produced a robust pulmonary inflammatory response with a similar peak right ventricular pressure (55 mmHg) [11]. Our previous studies indicated that the inflammatory response was not due to direct interactions between endothelial cells and the polystyrene microspheres, since cultures of vascular endothelial cells did not produce inflammatory signals in the presence of the microspheres [11]. Thus, it appears that pulmonary inflammation results from persistent blockage of the pulmonary circulation. Furthermore, this inflammatory response increased in a dose-dependent fashion and is most robust when the infused microsphere burden is sufficient to result in sustained pulmonary hypertension. These findings further support our conclusion that persistent vascular obstruction sufficient to induce sustained hypertension is required to induce a robust inflammatory response.

In our pilot work, we performed necropsies immediately after clot injection and found that the injected clot material does indeed embolize to the pulmonary arteries (Figure 2). Right ventricular pressures were acutely increased following clot embolization to approximately 55 mm Hg in Sprague Dawley rats and to approximately 45 mm Hg in the Copenhagen rats. We attempted to enhance the persistence of embolized clot material by pretreating the animals with an antifibrinolytic agent, tranexamic acid, and by adding calcium and thrombin to the tubing to enhance clot formation. We also "aged" the clot to enhance clot durability. Despite these measures, the both rat strains manifested highly active and efficient endogenous fibrinolysis as evidenced by the significant increase in D-dimer concentrations at 8 hours, the calculated clot lysis rates, and normalization of right ventricular pressures by 24 hours. A finding of potential significance, was that Copenhagen animals had a significantly lower clot lysis rate than Sprague-Dawley rats at 24 hours (p < 0.001) and a marginally lower rate at 5 days (p = 0.052). This finding supports the prior report by Cooley, et al of a prothrombotic tendency in the Copenhagen strain [9]. These animals also required significantly less clot (2.8 mg/100 gm vs. 22 mg/100 gm) to reach the goal right ventricular pressure. Preliminary attempts to increase the dosing with clot were fatal in Copenhagen rats. Being an inbred strain, the Copenhagen rats may be less robust, compared with the outbred Sprague Dawley rats. Alternatively, the difference in sensitivity may be due to the lower rate of clot lysis in the Copenhagen rats. The lower rate of clot lysis was reflected in the physiology of the Copenhagen animals, inasmuch as the right ventricular peak systolic pressure was slower to recover to baseline and only partially recovered from the peak value by the 24 hour period (Figure 3). Despite these observations, the Copenhagen animals still did not demonstrate a persistent clot burden sufficient to result in the sustained pulmonary hypertension that appears necessary to induce a robust inflammatory response.

## Conclusions

A minimally-invasive autologous clot model of moderate severity, demonstrated a significantly lower rate of clot lysis in the Copenhagen strain compared with Sprague-Dawley animals. Neither strain manifested the persistent pulmonary hypertension or lung inflammation that is seen with fixed-occlusion models, suggesting limited utility of the autologous clot-based modeling for the study of PE in rats. Persistent pulmonary vascular occlusion sufficient to result in sustained pulmonary hypertension appears necessary to induce robust lung inflammation after PE.

## Abbreviations

BAL: bronchoalveolar lavage; BCA: bicinchoninic acid; CINC: cytokine-induced neutrophil chemoattractant; MCP: monocyte chemoattractant protein; MPO: myeloperoxidase; PE: pulmonary embolism.

## Competing interests

The authors declare that they have no competing interests.

## Authors' contributions

MSR: Conducted some of the surgical procedures, oversaw data collection and participated in the writing of the manuscript. MAG: Conducted surgical procedures and collected in vivo data. NS: Conducted chemotaxis assays and chemokine ELISA assaysJAK: Participated in the planning of the study, interpretation of the results and participated in the writing of the manuscript. JAW: Participated in the planning of the study, data organization and graph production, interpretation of the results and participated in the writing and submission of the paper. All authors have read and approved the final manuscript.
